# Obesity-related parameters in carriers of some BDNF genetic variants may depend on daily dietary macronutrients intake

**DOI:** 10.1038/s41598-023-33842-4

**Published:** 2023-04-21

**Authors:** Urszula Miksza, Edyta Adamska-Patruno, Witold Bauer, Joanna Fiedorczuk, Przemyslaw Czajkowski, Monika Moroz, Krzysztof Drygalski, Andrzej Ustymowicz, Elwira Tomkiewicz, Maria Gorska, Adam Kretowski

**Affiliations:** 1 Department of Nutriomics, Clinical Research Centre, Medical University of Bialystok, Marii Sklodowskiej-Curie 24A, 15-276 Bialystok, Poland; 2 Clinical Research Support Centre, Medical University of Bialystok, Marii Sklodowskiej-Curie 24A, 15-276 Bialystok, Poland; 3 Department of Radiology, Medical University of Bialystok, Marii Sklodowskiej-Curie 24A, 15-276 Bialystok, Poland; 4 Department of Endocrinology, Diabetology and Internal Medicine, Medical University of Bialystok, Marii Sklodowskiej-Curie 24A, 15-276 Bialystok, Poland

**Keywords:** Obesity, Nutrition

## Abstract

Some common single-nucleotide polymorphisms of the brain-derived neurotrophic factor (BDNF) gene have been associated not only with the neurodegenerative diseases but also with some eating disorders. The aim of this study was to assess the possible differences in the obesity-related and glucose metabolism parameters between some BDNF genotypes’, that may depend on the daily energy and macronutrients intake. In 484 adult participants we performed the anthropometric measurements, body composition analysis, and body fat distribution. The daily dietary intake was assessed using the 3-day food intake diaries. Blood glucose and insulin concentrations were measured at fasting and during oral glucose tolerance tests. Moreover, the visceral adipose tissue/subcutaneous adipose tissue (VAT/SAT) ratio and homeostatic model assessment of insulin resistance were calculated. We noted that participants carrying the GG genotype had lower skeletal muscle mass and fat free mass (FFM) when carbohydrate intake was > 48%, whereas they presented higher fat-free mass (FFM), and surprisingly higher total cholesterol and LDL-C concentrations when daily fiber intake was > 18 g. Moreover, in these subjects we noted higher waist circumference, BMI, and fasting glucose and insulin concentrations, when > 18% of total daily energy intake was delivered from proteins, and higher VAT content and HDL-C concentrations when > 30% of energy intake was derived from dietary fat. Our results suggest that glucose homeostasis and obesity-related parameters in carriers of some common variants of BDNF gene, especially in the GG (rs10835211) genotype carriers, may differ dependently on daily energy, dietary macronutrients and fiber intake.

## Introduction

One of the most significant risk factor of glucose homeostasis disturbances and type 2 diabetes (T2DM) is obesity. Excess body weight is a major risk factor also for the other non-communicable diseases^1^ including cardiovascular disease (CVD), lipid disorders, and certain types of cancers. It has been estimated that 44% of cases of T2DM, 23% of ischemic heart disease cases, and 7–41% of certain cancers are attributable to overweight and obesity^2,3^. Obesity, mostly, is a result of following unhealthy dietary patterns and a sedentary lifestyle, but a genetic component may also play an important role, especially by its interaction with the abovementioned environmental factors^4^. A strong genetic component underlying individual variations in body weight may determine a human’s response to ‘obesogenic’ environmental factors, and in twin and family studies, it has been estimated that the heritability of obesity is between 40 and 70%^5,6^.

Genome-wide association studies (GWAS) have so far identified > 1000 loci that may affect body weight^5,7,8^ and confirmed a strong heritability of T2DM^9,10^. Exploring the complex of polygenic factors that influence body weight supports the central role of the brain in regulating energy balance, highlighting the roles of the leptin–melanocortin pathway and TrkB–BDNF (tropomyosin receptor kinase B–brain-derived neurotrophic factor) signalling^4^. Polymorphisms within those pathways produce subtle results affecting body weight, and they may also be associated with other obesogenic factors. BDNF is a member of the neurotrophin family and an important initiator in neurodevelopmental processes, playing primary roles in the regulation of neuronal survival and differentiation in both the peripheral and central nervous systems^11^. The BDNF gene has been already associated in the GWAS with obesity^8^. It is highly expressed in the hypothalamus, which is a center of appetite, suggesting that it may affect energy balance, and it has been reported that this expression may also be dependent on nutritional status^12^, diet energy or omega-3 fatty acids^13^. The Val/Met and Met/Met polymorphisms within the BDNF gene are associated with 30% higher risk of anorexia nervosa and bulimia (paroxysmal/laxative), which indicates that the BDNF Val66Met polymorphism is strongly associated with eating disorders^14^.

Even if obesity and T2DM have strong genetic component, they should not be considered as only genetic disorders, because as mentioned above also the other, especially dietary factors play very important role as well. Among all macronutrients, carbohydrates are one the most controversial nutrient^15^. Carbohydrates are one of the major components of most usual diets^16^ and have been linked to overweight and T2DM for many decades^17^. Nevertheless, there is no consistent evidence that carbohydrates determinate the present levels of global obesity^18–20^. One of the reasons of discrepancies between results of studies evaluating the influence of diet on metabolic parameters may be the effect of genetic variations^21^, what we have also observed in our previously published studies^22–24^,and what has been reported also by Zhou JY et al.^25^.

In pursuance of the studies, some common polymorphisms of the BDNF gene, such as rs6265, rs4923461, rs10501087, rs10835211, rs12291063, and rs1488830 seem to be genetic risk factors for obesity^26,27^. Interesting results were obtained by Sandholt CH et al., who noted that the obesity risk allele of BDNF rs4923461 may present a protective role against T2DM^28^. Additionally, BDNF polymorphisms may be differentially associated with obesity, dependent on sex: the rs6265 GG genotype has been correlated with obesity in men but with a lower body mass index (BMI) in women^29^. A study by Duˇs´atkov´a L et al.^30^ showed that rs925946 may interact with dietary factors, which may affect body weight^31,32^.

Taking into consideration the studies mentioned above conducted in human and animal models, we hypothesized that the relation between some common SNPs of the BDNF gene (rs6265, rs4923461, rs10501087, rs10835211) and obesity-related and glucose homeostasis parameters may be dependent on dietary factors. With strong possibility, we could suppose that our study is one of the first to investigate if carriers of some common BDNF SNPs may present different results of investigated parameters dependently on daily energy, fibre and macronutrients intake.

## Materials and methods

### Ethics statement

All of the research procedures in this study were in agreement with of the ethical standards for human studies and with the Declaration of Helsinki revised in 1983. All participants singed informed consent before an enrollment in the study. The study protocol and all study procedures were approved by the local Ethics Committee of Medical University of Bialystok, Poland (R-I-002/35/2009).

### Participants

In this study, we included data collected from 484 subjects of the 1000PLUS Cohort Study that included a Caucasian population of Polish origin (study registered at www.clinicaltrials.gov as NCT03792685). Subjects were described in detail previously^22–24^, and the enrolment process is presented in the Supplementary Materials. In the analysis, we included only those participants who properly completed food diaries. Subjects who did not fully complete 3-day food diaries or did not provide food portions were not included in this study. Volunteers who used to take medicines or diet supplements, in particular, anti-diabetic, weight loss, cholesterol-lowering, or any other medication that could affect body weight, body fat content, blood glucose, and other estimated parameters, were not included in the analysis. Moreover, from our analysis, we excluded subjects with a history of bariatric surgery or of endocrine, gastrointestinal, renal, hepatic, metabolic, immunological, or psychiatric disorders, along with subjects who followed a special diet (e.g., vegetarian, vegan, Dukan diet, fasting), which could have an impact on the investigated parameters.

### Anthropometric measurements and body composition analysis

In all study participants, we performed body weight and height measurements, waist and hip circumference measurements, and body composition analysis (InBody 220, Bio-Space, Korea), including the total body fat content, fat-free mass (FFM), and skeletal muscle mass (SMM). Visceral adipose tissue (VAT) and subcutaneous adipose tissue (SAT) contents were measured via bioelectrical impedance analysis (Maltron 920-2 BioScan, Maltron International Ltd, UK). All of the measurements were performed in the morning, after an overnight fast for 8–12 h.

### Blood collection and biochemical analysis

#### Oral glucose tolerance test (OGTT)

Subjects underwent OGTTs following the World Health Organization (WHO) recommendations, with a dose of 75 g oral glucose load. All the participants were instructed to fast for 8–12 h before the OGTT and to not restrict dietary carbohydrate intake for at least 3 days before the test. Blood samples were collected at fasting and at 30, 60, and 120 min after the glucose load.

### Biochemical analysis

From the collected blood samples, we evaluated the blood glucose, insulin, high-density lipoprotein (HDL), low-density lipoprotein (LDL), total cholesterol, and triglyceride (TG) concentrations, as well as hemoglobin A1c (HbA1c). The samples were prepared following the laboratory kit instructions of producer. Serum insulin concentrations were assessed by immunoradiometric assay (INS-Irma, DIA Source S.A., Belgium; using Wallac Wizard 1470 Automatic Gamma Counter, PerkinElmer Life Sciences, Turku, Finland). The plasma glucose concentrations were assessed by the hexokinase enzymatic method (using Cobas c111, Roche Diagnostics Ltd., Switzerland). The lipid profile including total cholesterol, LDL-C, HDL-C, and TG concentrations were evaluated by the enzymatic colorimetric assays using commercially available laboratory kits (Cobas c111, Roche Diagnostic Ltd., Switzerland). HbA1c levels were measured by a HPLC (high-performance liquid chromatography) method (using D-10 Hemoglobin Testing System, Bio-Rad Laboratories Inc., Hercules, CA, USA; and Bio-Rad, Marnes-la-Coquette, France).

### Dietary intake and daily physical activity analyses

The daily dietary intake among participants was evaluated using completed 3-day food intake diaries. Participants estimated food portion sizes using the color photo albums with the portion sizes. In addition, participants were instructed how to weigh the food portions, if possible. The total energy, carbohydrate, fiber, protein, and fat intakes were estimated using a Dieta 6.0 software (provided by the National Food and Nutrition Institute, Warsaw, Poland). Dieta software was developed and is being continuously up-dated, and it is a scientific tool which is commonly used to calculate the nutritional value of diet.

In order to evaluate the differences in studied parameters between carriers of investigated BDNF genetic variants that may be dependent on dietary factors, the study subject were divided into two quantiles groups based on their average daily kcal, carbohydrate, protein, fat, and fiber intakes as mentioned above: lower and higher quantiles of daily energy intake (≤ 1800 and > 1800 daily kcal intake, respectively), lower (≤ 18%) and higher (> 18% of total energy intake) quantiles of dietary protein intake; lower (≤ 48%) and higher (> 48% of total energy intake) quantiles of dietary carbohydrate intake, lower (≤ 30%) and higher > 30% of total energy intake) quantiles of dietary fat intake, and lower (≤ 18 g) and higher (> 18 g daily intake) quantiles of dietary fiber intake.

To evaluate daily physical activity level, the IPAQ-LF (International Physical Activity Questionnaire—Long Form) was used^33^, and participants were stratified to one of the groups: with a low, moderate, or high level of daily physical activity, following the LPAQ-LF instructions.

#### Genetic analyses

Based on the available scientific literature^26^, we selected and genotyped 4 BDNF polymorphisms that are considered genetic risk factors for obesity: rs6265, rs4923461, rs10501087, and rs10835211. DNA was extracted from the peripheral blood leukocytes by a classical salting-out method. The analyzed SNPs were genotyped with a TaqMan SNP technology from a ready-to-use human assay library (Applied Biosystems, MA, USA) using a high-throughput system (Open Array, Life Technologies, CA, USA). We performed SNP analysis in duplicate, according to the manufacturer’s instructions. As a negative control we used a sample without a template to detect possibly caused by contamination false-positive signals.

### Calculations

BMI was calculated using the standard formula: body weight (kg) dividing by height (m) squared. The WHR (waist–hip ratio) was calculated by dividing waist circumference (cm) by hip circumference (cm). The same method was used to calculate the VAT/SAT ratio. The HOMA-IR (homeostatic model assessment of insulin resistance) was estimated following the formula: (fasting plasma glucose concentration (mmol/L)) × (fasting insulin concentration (μU/mL))/22.5. A metabolic equivalent (MET) was calculated using the following formula: (MET level) × (minutes of activity) × (events per week).

### Statistical analysis

The statistical analysis performed in this study aimed to compare the differences between numerical and categorical variables in participants divided into two groups based on their median daily intake of kcal, carbohydrates, protein, fat, and fiber. The numerical data were presented as the number of observations (N), mean, and standard deviations (SD), while the categorical data were presented as the number of observations and frequency (%). The participants were divided into two groups based on their median daily intake of these parameters, and the differences between the numerical variables and dietary groups were analyzed using the Kruskal–Wallis test. This test was chosen because it can be used to compare differences between two or more independent groups with non-normal distributions, which was the case in this study. The Kruskal–Wallis test was followed by Dunn’s post-hoc test and Holm* p*-value adjustment, which were used to perform multiple pairwise tests and adjust for multiple grouping variables. The categorical variables were compared using a chi-squared test, which is a commonly used test for comparing frequency distributions between two or more categories.

A significance level of < 0.05 was set for all 2-sided tests, meaning that any results that were found to be significant at this level were considered to be statistically significant. The calculations for all statistical tests were performed in the R version 4.2.2.

In conclusion, the statistical analysis performed in this pilot study aimed to determine the differences between the clinical parameters in participants divided into two groups based on their median daily intake of kcal, carbohydrates, protein, fat, and fiber. The results of the statistical tests were used to identify any significant differences between the groups. These results provide valuable information for understanding the relationships between different variables and can be used to make important decisions in further research or practical applications.

## Results

The linkage disequilibrium (LD) pairwise test showed that the investigated loci are in strong LD (Table 1); therefore, we present results for one of them—rs10835211.

**Table 1:** The results of linkage disequilibrium analysis.

Investigated SNPs	*BDNF*_rs10835211	*BDNF_* rs4923461	*BDNF_* rs6265
***BDNF*_rs10501087**			
D’	0.9341	0.9838	0.8228
*p*-value	< 0.0001	< 0.0001	< 0.0001
***BDNF*_rs10835211**			
D’	X	0.9133	0.5407
*p*-value	X	< 0.0001	< 0.0001
***BDNF*_rs4923461**			
D’	X	X	0.7785
*p*-value	X	X	< 0.0001

### Study group characteristics

The characteristics of the study population stratified by the investigated genotypes are presented in Table 2.

**Table 2:** The study group characteristics stratified by rs10835211 genotypes.

rs10835211	Whole study group	study group stratified by rs10835211 genotypes			
		AA	AG	GG	*p*-value
N	484	16	147	321	–
Female/Male (n)	271/219	9/7	78/70	173/153	–
Female/Male (%)	55.3/44.7	55.6/44.4	52.7/47.3	53/47	–
Genotype frequency	–	0.04	0.34	0.62	> 0.05
Age (years)	42.8 (14.8)	43.39 (13.60)	40.11 (14.45)	40.19 (14.31)	0.43
BMI (kg/m2)	27.4 (6.6)	29.68 (7.03)	28.82 (7.02)	28.06 (6.44)	0.64
< 25.0 (n;%)	167 (34.5%)	6 (37.5%)	57 (38.8%)	104 (32.4%)	0.58
25.0–29.9 (n;%)	168 (34.7%)	7 4 (25.0%)	40 (27.2%)	124 (38.6%)	
≥ 30.0 (n;%)	149 (30.7%)	6 (37.5%)	50 (34.0%)	93 (29.0%)	
Total body fat content (kg)	26.8 (13.9)	30.21 (13.85)	27.86 (14.25)	26.36 (13.57)	0.29
Total body fat content (%)	31.7 (9.6)	34.16 (8.42)	31.82 (9.72)	31.02 (9.49)	0.11
Waist circumference (cm)	95.5 (17.1)	98.33 (17.34)	97.06 (18.58)	95.32 (16.36)	0.56
Hip circumference (cm)	103.1 (12.8)	105.68 (12.68)	104.10 (13.52)	102.60 (12.32)	0.41
WHR	0.922 (0.09)	0.93 (0.09)	0.92 (0.08)	0.92 (0.08)	0.98
VAT (cm3)	108.2 (77.6)	109.95 (72.61)	106.23 (84.74)	108.54 (78.96)	0.32
VAT (%)	36.9 (12.3)	33.13 (8.23)	35.70 (12.26)	37.78 (11.94)	0.08
SAT (cm3)	168.2 (82.1)	202.63 (82.43)	174.36 (86.00)	163.15 (79.15)	0.07
SAT (%)	63.8 (11.9)	66.86 (8.23)	64.29 (12.25)	62.24 (11.90)	0.08
VAT/SAT ratio	0.66 (0.43)	0.52 (0.18)	0.63 (0.41)	0.68 (0.46)	***0.045***
Total cholesterol (md/dl)	194.2 (42.9)	190.50 (29.83)	198.42 (46.22)	193.18 (42.35)	0.61
HDL (mg/dl)	60.7 (14.9)	60.86 (15.45)	59.07 (15.09)	60.54 (14.78)	0.56
LDL (mg/dl)	111.0 (39.9)	107.02 (29.75)	116.18 (43.42)	110.33 (39.61)	0.50
TG (mg/dl)	111.3 (68.4)	113.07 (53.19)	126.18 (137.00)	111.53 (67.85)	0.37
Fasting glucose concentration (mg/dl)	93.7 (13.5)	94.02 (11.20)	94.82 (17.12)	93.30 (11.45)	0.76
Glucose concentration during OGTT (mg/dl) at 30 min	147.3 (36.4)	141.53 (18.97)	148.82 (39.72)	146.27 (35.47)	0.89
Glucose concentration during OGTT (mg/dl) at 60 min	131.3 (48.6)	131.06 (27.61)	134.52 (53.46)	129.59 (47.19)	0.56
Glucose concentration during OGTT (mg/dl) at 120 min	99.8 (34.7)	102.44 (29.14)	102.79 (42.70)	97.10 (31.15)	0.21
Fasting insulin concentration (IU/mL)	12.1 (8.7)	12.44 (7.48)	13.64 (11.33)	11.70 (8.59)	0.25
Insulin concentration at 30 min of OGTT (IU/mL)	73.9 (45.7)	75.55 (46.86)	74.19 (46.81)	73.03 (45.59)	0.99
Insulin concentration at 60 min of OGTT (IU/mL)	78.1 (61.7)	90.78 (71.58)	79.04 (57.97)	75.46 (64.30)	0.17
Insulin concentration at 120 min of OGTT (IU/mL)	43.5 (40.8)	56.27 (47.08)	44.13 (37.42)	41.16 (41.61)	***0.007***
Daily total energy intake (kcal)	1822 (716.6)	1839.03 (604.48)	1730.38 (674.26)	1807.82 (701.47)	0.08
Daily energy from carbohydrates intake (%)	47.6 (8.6)	44.74 (9.38)	47.90 (8.13)	47.70 (8.71)	0.22
Daily energy from protein intake (%)	18.6 (4.8)	17.90 (3.59)	19.37 (4.21)	18.75 (5.05)	***0.035***
Daily energy from fat intake (%)	31.2 (7.5)	34.87 (7.59)	30.65 (7.49)	31.30 (7.44)	0.058
Daily fiber intake (g)	18.7 (8.3)	18.49 (7.38)	18.84 (8.11)	18.14 (7.04)	0.78
Daily physical activity level (%)					
Low	12 (2.5%)	14.81 (36.20)	9.02 (28.7)	5.27 (22.37)	0.74
Moderate	95 (19.6%)	11.11 (32.03)	19.67 (39.83)	21.29 (40.98)	0.07
High	377 (77.9%)	74.07 (44.66)	71.31 (45.32)	73.44 (44.21)	0.81

Data are presented as the arithmetic mean values and standard deviations (SDs), or numbers and percentage if indicated.

Standard deviations are in bolditalic.

## The BDNF rs10835211 polymorphism, obesity, glucose homeostasis parameters, and lipid profiles, dependently on dietary factors

### Dietary stratification groups

The numbers of participants in every study group divided by the dietary and genotypes stratifications are presented in Table 3.

**Table 3:** The study group stratified by genotypes ans daily dietary intake.

Genotype and dietary stratification	AA, n = 16	AG, n = 147	GG, n = 321
Kcal ≤ 1800 l (n)	8	91	186
Kcal > 1800 l (n)	8	56	135
Fat ≤ 30% (n)	5	73	179
Fat > 30% (n)	11	74	142
Protein ≤ 18% (n)	9	61	171
Protein > 18% (n)	7	86	150
Carbohydrates ≤ 48% (n)	11	71	157
Carbohydrates > 48% (n)	5	76	164
Fiber ≤ 18 g (n)	9	70	170
Fiber > 18 g (n)	7	77	151

We did not notice any significant differences in physical activity between studied groups. Due to the large number of analysis performed we present results only for statisticaly significant differences.

### Diet energy stratification

**Figure 1:**
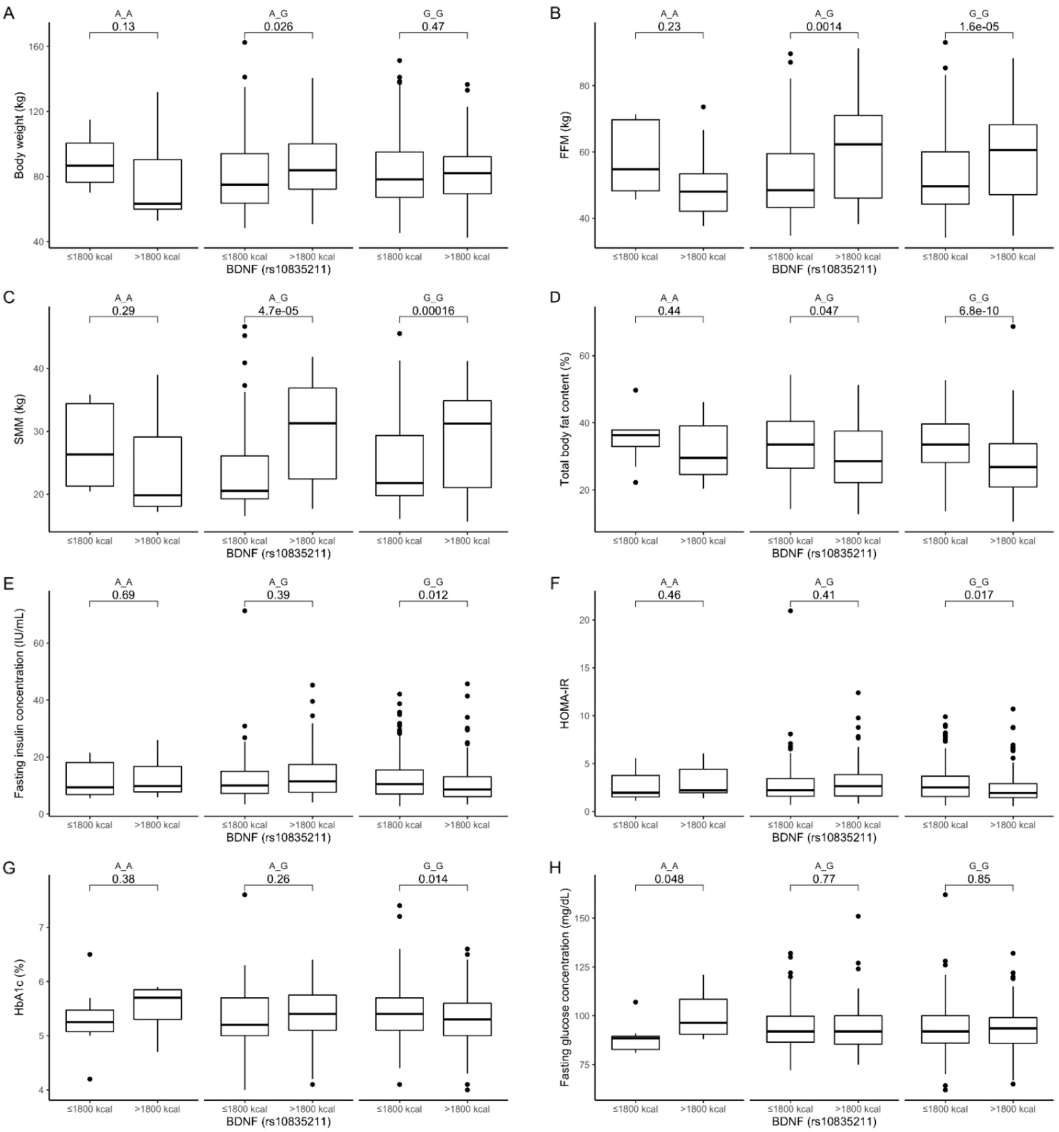
The (**A**) body weight (kg); (**B**) FFM (kg); (**C**) SMM (kg); (**D**) total body fat content (%); (**E**) fasting insulin concentrations (IU/mL); (**F**) HOMA-IR; (**G**) HbA1c; and (**H**) fasting glucose concentration (mg/dL) in BDNF rs10835211 genotype carriers, stratified by daily energy intake (≤ 1800 kcal and > 1800 kcal).

In the AG genotypes, in subjects from the lower daily energy intake quantiles, we noted significantly lower body weight (Fig. 1A), while in the AG and GG genotype carriers stratified to the same group daily energy intake group we observed lower FFM (Fig. 1B), and SMM (Fig. 1C), whereas the total body fat percentage was significantly higher (Fig. 1D) compared to that of subjects from the higher quantiles of daily energy intake. Surprisingly, the GG genotype carriers stratified to the lower quantiles of daily energy intake showed significantly higher fasting insulin concentrations (Fig. 1E), higher HOMA-IR (Fig. 1F), and higher HbA1c (Fig. 1G), as compared to the same genotype carriers stratified to the upper quantiles of daily energy intake. In the AA homozygotes stratified to the lower quantiles of daily energy intake, we noticed lower fasting glucose concentrations (Fig. 1H). We did not observe any significant differences in lipid parameters between groups stratified by a daily energy intake (data not shown).

### Dietary carbohydrate intake stratification

**Figure 2:**
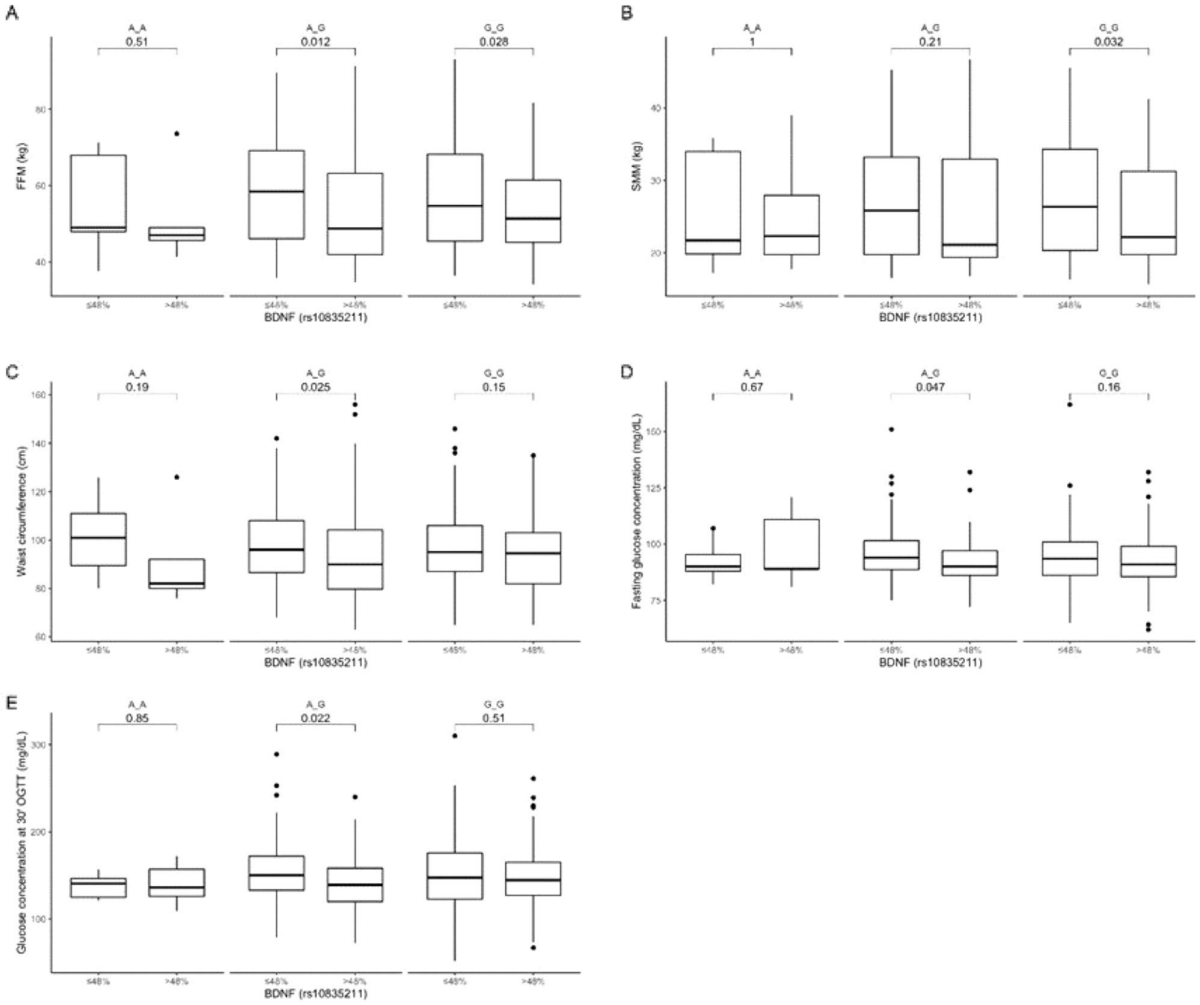
The (**A**) FFM (kg); (**B**) SMM (kg); (**C**) waist circumference (cm); (**D**) fasting glucose concentration (mg/dl); and (**E**) glucose concentration at 30’ of the OGTT in BDNF rs10835211 genotype carriers, stratified by daily carbohydrates intake (≤ 48% and > 48% of total daily energy intake).

GG homozygotes with carbohydrate intake higher than the median had lower FFM (Fig. 2A) and SMM (Fig. 2B), whereas in carriers of the AG genotype from the upper quantiles of carbohydrate intake, we noted significantly lower waist circumference (Fig. 2C), glucose concentrations at fasting (Fig. 2D) and at 30 min on the OGTT (Fig. 2E). We did not observe any significant differences of studied parameters in the AA genotype carriers.

### Dietary protein intake stratification

**Figure 3:**
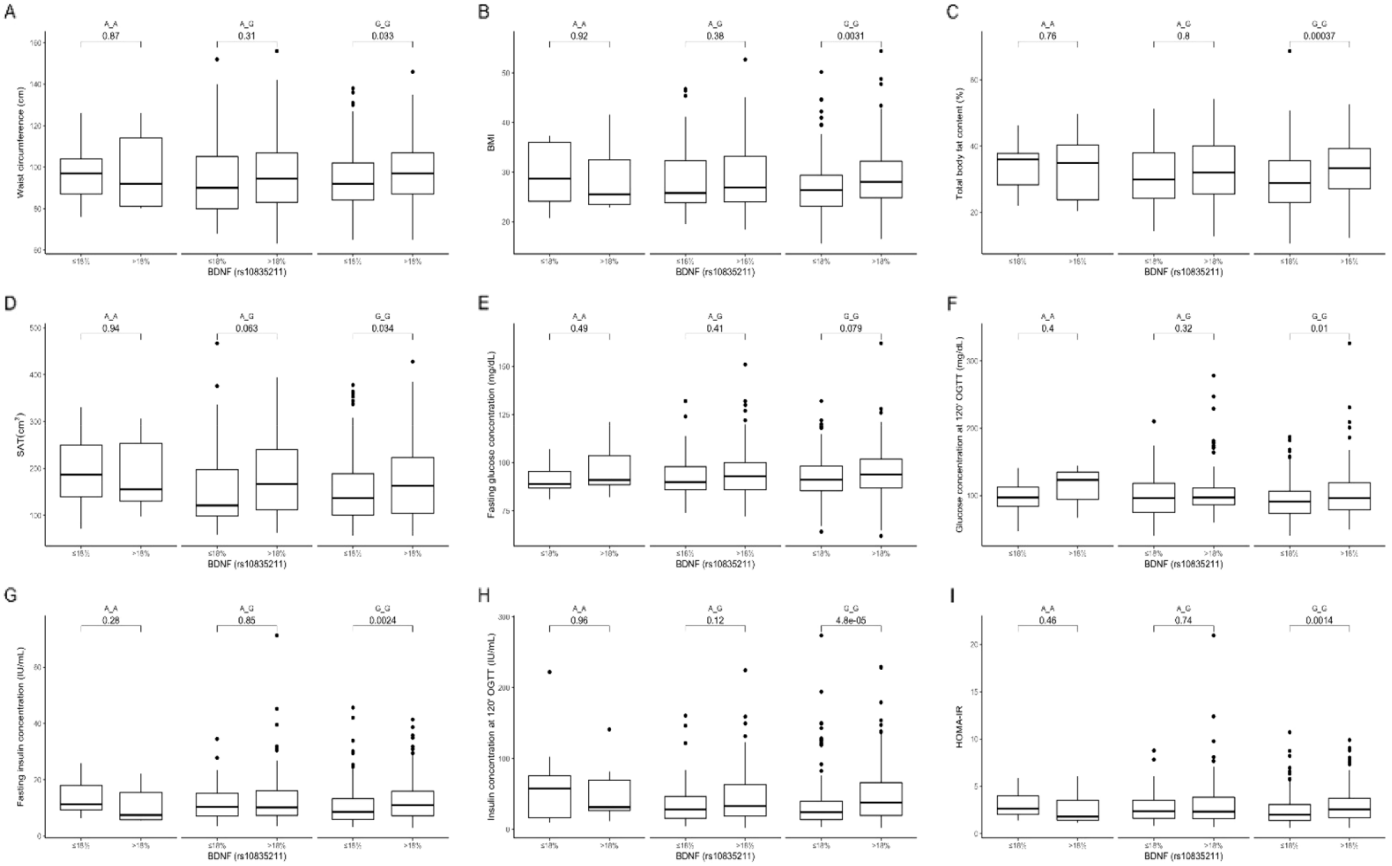
The (**A**) waist circumference (cm); (**B**) BMI (kg/m2); (**C**) total body fat content (%); (**D**) SAT (cm3); (**E**) fasting glucose concentration (mg/dl); (**F**) glucose concentration (mg/dl) at 120’ OGTT; (**G**) fasting insulin concentration (IU/mL); (**H**) insulin concentration at 120’ on the OGTT; and (**I**) HOMA-IR in BDNF rs10835211 genotype carriers stratified by daily protein intake (≤ 18% and > 18% of the total daily energy intake).

Our results showed that GG genotype subjects with protein intake > 18% of their total daily energy intake had higher waist circumference (Fig. 3A), BMI (Fig. 3B), total body fat content (Fig. 3C), and volume of SAT (Fig. 3D) compared to the same genotype carriers from the lower quantiles of protein intake. In the GG genotype carriers, with dietary protein higher than 18% of daily energy intake, we noted a tendency to higher glucose concentrations at fasting (Fig. 3E) and significantly higher glucose concentration at 120’ on the OGTT (Fig. 3F), as well as significantly higher insulin concentrations at fasting (Fig. 3G) and at 120’ on the OGTT (Fig. 3H). GG genotype participants stratified to the upper protein intake quantiles also showed significantly higher HOMA-IR (Fig. 3I). We did not observe any differences dependent on dietary protein intake in AA homozygotes and AG heterozygotes or any differences in lipid profile in any of the studied genotypes.

### Dietary fat intake stratification

**Figure 4:**
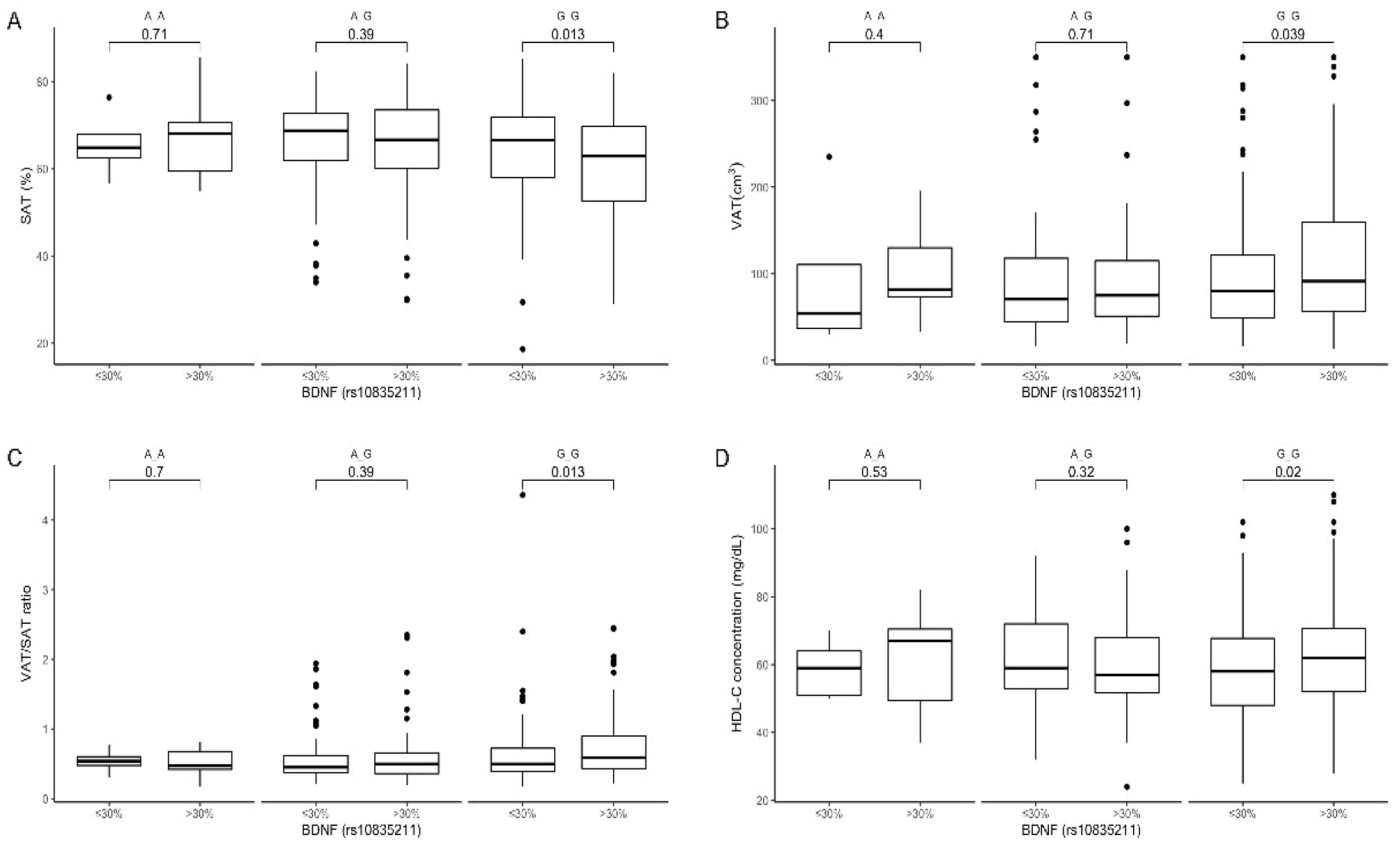
The (**A**) SAT (cm3); (**B**) VAT (cm3); and (**C**) VAT/SAT ratio (**D**); HDL-C concentration (mg/dL) in BDNF rs10835211 genotypes carriers stratified by daily fat intake (≤ 30% and > 30% of total daily energy intake).

The GG genotype participants from the upper quantiles of fat intake showed lower SAT (Fig. 4A) but higher VAT content (Fig. 4B), as well as higher VAT/SAT ratio (Fig. 4C). Surprisingly, GG genotype carriers with daily fat intake higher than the median also presented significantly higher HDL-C concentrations (Fig. 4D). We did not observe any differences dependent on the dietary fat intake in AA homozygotes and AG heterozygotes, nor did we observe any differences in glucose homeostasis in any of the studied genotypes of rs10835211.

### Dietary fiber stratification

**Figure 5:**
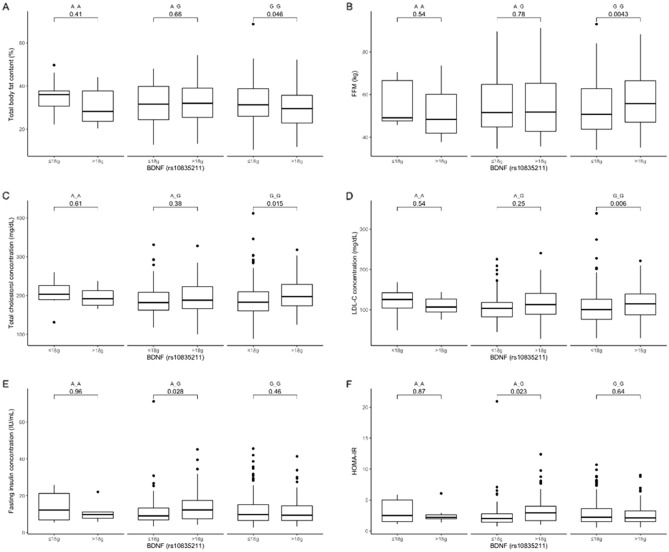
The (**A**) total body fat content (%); (**B**) FFM (kg); (**C**) total cholesterol concentration (mg/dL); (**D**) LDL-C concentration (mg/dL); (**E**) fasting insulin concentration (IU/mL); and (**F**) HOMA-IR in BDNF rs10835211 genotype carriers stratified by daily dietary fiber intake (≤ 18 g and > 18 g).

Carriers of the GG genotype from the upper quantiles of fiber intake presented lower total body fat content (Fig. 5A) and higher FFM (Fig. 5B), but also higher total cholesterol (Fig. 5C) and LDL-C concentrations (Fig. 5D). In the AG genotype carriers stratified to the upper quantiles of fiber intake, we observed higher fasting insulin concentration (Fig. 5E), as well as higher HOMA-IR (Fig. 5F). We did not observe any differences dependent on dietary fiber intake in the AA genotype carriers.

## Discussion

To the best of our knowledge, our study is the first to explore the link between anthropometric parameters, glucose, and lipid homeostasis, and BDNF rs10835211 genetic variants depending on diet energy and macronutrient stratification. In our cross-sectional study, we investigated the possible differences in obesity-related parameters dependently on daily energy and macronutrients intake in carriers of four BDNF genetic variants (rs6265, rs4923461, rs10501087, rs10835211). Investigated SNPs we found to be in strong LD; therefore, we demonstrated results for one of them, rs10835211. We analyzed the link between daily energy, macronutrients dietary carbohydrates, protein, fat, and fiber intake stratification and the occurrence of metabolic differences in the carriers of common polymorphisms in rs10835211 of the BDNF gene.

We noted the highest VAT/SAT ratio but the lowest insulin concentrations at 120 min of the OGTT test in the GG genotype carriers. We have observed that without any differences in BMI and total body fat content between the studied genotypes. A study from Mexico^34^ found an existing association between the risk of overweight and obesity (defined based on BMI) and the GG genotype of rs6265, which we found to be in strong LD with the rs10835211, however, that study did not include body fat distribution, so we were not able to compare the obtained results. Nevertheless, the highest VAT/SAT ratio that we noted suggests that GG genotype carriers may be at higher cardiometabolic risk, since a higher VAT/SAT ratio (which indicates the propensity to store fat viscerally versus subcutaneously) was found to be an independent cardiometabolic risk factor, above and beyond BMI and visceral fat content^35^. The lowest insulin concentrations at 120 min of OGTT in the GG genotype individuals in spite of higher VAT/SAT ratio were surprising as well. However, as it was noted by Gastaldelli A. et al.^36^ hyperinsulinemia is appropriate for the degree of insulin resistance regardless of obesity and abdominal fat distribution, and none of the parameters describing beta cell function was noted to be independently associated with excess VAT content in non-diabetic individuals, what could be an explanation of results observed in our study. Moreover, we noted very interesting findings when we stratified subjects by daily energy intake. Participants with the AG genotype who consumed more than 1800 kcal/day showed higher body weight but, surprisingly, the AG as well as GG genotype carriers, showed lower total body fat content, and the higher body weight was a result of higher FFM and SMM, when compared to subjects carrying the same genotype but who consumed less than 1800 kcal/day. It is worth noting that we did not observe any significant differences between the studied genotype carriers in terms of physical activity levels that could explain the lower body fat and higher FFM content in spite of higher energy intake, which suggests that this body composition may be independent of physical activity. Moreover, subjects carrying the GG genotype, who reported higher than median daily energy intake, presented lower fasting insulin concentrations, lower HbA1c, and lower HOMA-IR levels. What is also interesting is that we did not observe any significant differences in obesity-related parameters that would be diet-dependent in the AA genotype carriers, except higher fasting glucose concentrations, when they were stratified to the higher daily energy intake quantiles. Daily JW et al.^37^ noted the opposite results: non-diabetic subjects with the GG genotype in rs6265 (Val/Val) with higher daily energy intake had a higher risk of developing T2DM, but this study included a Korean population; therefore, possible ethnic differences should also be considered^38^.

When we analyzed dietary carbohydrate intake, surprisingly, we found that GG homozygotes with daily carbohydrate intake higher than the median presented lower FFM and lower skeletal muscle mass. These results are in contrast with our previous reports on the benefits of more than 48% of the total daily energy provided from carbohydrates, in carriers of some SNPs within the other genes^22–24^. In the AG genotype carriers, we observed lower FFM and waist circumference, along with lower glucose concentrations at fasting and after 30 min of glucose load. We did not observe any differences in studied parameters that might be dependent on carbohydrate intake in the carriers of the AA genotype, what suggest that daily carbohydrate intake may not have any impact on the glucose homeostasis in these subjects. Our observations may explain the differences between results obtained from other studies, which investigated the associations between dietary carbohydrates and obesity or T2DM, while one of them suggested that low-carbohydrate diets may be a very effective treatment option for subjects with T2DM and obesity^39^, whereas another suggested that diets in which carbohydrates make up more than 50% of total daily energy intake may have a protective effect on glucose–insulin metabolism^40^. As we noticed in our study, the results may also differ depending on the BDNF genotype. Nevertheless, it should also be considered that we have not analyzed the quality of the carbohydrates consumed. And it has been established that diet with a high proportion of ultra- processed foods, rich in refined carbohydrates and saturated fatty acids; increases energy intake, leading to weight gain and disturbances in carbohydrate metabolism^41^.

Additionally, we noted that also dietary protein intake may have an important impact on obesity and glucose-homeostasis-related parameters, but only in GG carriers. In these participants stratified to protein intake above > 18% of their total daily energy, we noted higher waist circumference, BMI, total body fat content, SAT volume, lower insulin sensitivity with higher glucose and insulin concentrations especially after glucose load. It has been suggested that dietary protein intake may be beneficial for body weight control by increasing satiety, increasing diet-induced thermogenesis, and increasing or maintaining FFM^42^. On the other hand, several years of observational study have indicated that dietary protein content above the physiological norm is associated with weight gain^43^. Our results may suggest that protein intake at > 18% of total daily energy by carriers of the GG genotype may have an adverse impact on the abovementioned obesity-related and glucose homeostasis parameters, which are strong predictors of T2DM^44^. A link between BDNF genetic variants, protein intake > 13%, and T2DM development was also already noted by Daily JW et al.^37^. Some of our previous analyses also showed that dietary protein intake above 18% may be related to glucose homeostasis in the carriers of some SNPs in the FTO^22^ and TCF7L2 genes^23^.

We also noticed that the GG genotype carriers stratified to the upper quantiles of dietary fat intake presented lower SAT but higher VAT content and higher VAT/SAT ratio. Moreover, we observed in these subjects lower FFM and SMM, but, surprisingly, higher HDL-C concentration. the relationship between BDNF SNPs and the lipid profile has been already noted: rs6265 A (Met) allele carriers were observed to have lower HDL-C concentration compared to non-A allele carriers^45^. It was postulated that the AA (Met/Met) genotype may be responsible for lower BDNF protein synthesis^46^. Presumably, the BDNF protein acts on the hypothalamus neurons, leading to the stimulation of the sympathetic nervous system, including the expenditure of energy and lipids^47^. Several studies have shown that VAT may play a pivotal role in regulating cholesterol metabolism, especially HDL-C^48,49^, and an abnormal distribution of adipose tissue indicates the disturbance of free fatty acid metabolism. The VAT is also responsible for the secretion of pro-inflammatory cytokines and correlates with a lower concentration of HDL-C^50^, which is contrary to our observations. We must underline that we did not analyze the source or the composition of dietary fat, and it has been reported that the percentage of fat in the diet may not play a crucial role because the ratio of saturated, unsaturated, and polyunsaturated fatty acids may be important as well^51^. This could explain our observations, and further investigation is needed.

Our study suggests that also dietary fiber may have a positive impact on body composition in GG genotype carriers. Even though we did not observe any differences in body weight, we noted lower total body fat content and higher FFM when daily fiber intake was > 18 g/day in the GG genotype carriers. The positive effects of dietary fiber are well known^52^, and high fiber intake may be associated with greater appetite suppression and, therefore, reduced energy intake^53^. However, in our study, we also noted an unexpected observation: the carriers of the GG genotype stratified to the high-fiber-intake quantiles presented higher LDL and total cholesterol levels, while AG carriers showed higher fasting insulin concentrations and higher HOMA-IR values. This was a very surprising finding, and we hypothesize that it might be associated with the source of dietary fiber, which we did not analyze in our study. It is known that high dietary fiber intake is associated with a reduced risk of T2DM^52^. However, the health outcomes of dietary fiber intake may be related to the source of fiber^54^. Refined fiber may show only a small or no effect on glycemic and lipid parameters^55^. It has been observed that higher consumption of wheat fiber increases the production of short-chain fatty acids (SCFA), which stimulate the secretion of Glucagon-like peptide-1 (GLP-1)^54^. However, in insulin resistance, this mechanism may be disrupted^56^; therefore, it seems that it might not be translated into the AG genotype carriers, who presented significantly higher HOMA-IR indices. We did not find any study that investigated the effect of dietary fiber consumption dependent on the genetic variants of BDNF rs10835211. However, an association between other genetic risk variants, dietary fiber intake, HbA1c levels, and the presence of T2DM was previously observed^57^.

Our results come from a preliminary study with some limitations; therefore, they should be interpreted with caution, and needs to be replicated in another study population. First of all, it was a cross-sectional study so that we could identify the differences in the investigated parameters between studied diet-stratified groups but not causes and effects. An intervention study may offer more reliable information about the genetic effects and diet interactions and it should be considered to be conducted. Moreover, we divided study population dependently on the carrying of few SNPs in one gene that may have a subtle but not large contribution to obesity development, since, as we mentioned above, obesity is a complex, multifactorial disease in which many pathways and other factors are involved. We must underline also the fact that due to relatively small sample size we did not divide our study group by the sex, and females and males have different body fat content, and different proportions of SAT and VAT volumes. Females may have also lower daily kcal intake than males. The genotypes in our study group did not differ in sex and total energy intake, so it might not have an impact on the general final results, nevertheless, all of these needs attention, and should be considered in further studies and analysis. It is needed to mention as a limitation the fact that to assess the visceral and subcutaneous fat contents we used a bioelectrical impedance method, but as it was reported this method show a high correlation coefficient with results of visceral fat area measured by X-ray computed tomography^58^. Subsequently, the daily dietary nutrient intake was assessed by a self-assessment using 3-day food diaries, and the daily macronutrient intake could be over- or underreported. Nevertheless, this method is acceptable and commonly used to assess the energy and nutritional value of a diet in large populations^59^. The other important limitation is the fact that we did not analyze the quality of macronutrients, sources of dietary fiber and dietary fats, nor did we analyze their composition, which could explain some of our observations. Additionally, an important limitation is the small number of volunteers in the group carrying the AA genotype variant, which was due to the genotype’s low frequency in the general population. On the other hand, it might be the reason for the lack of significance in some results; therefore, more studies in larger and more diverse populations are needed to validate our findings.

## Conclusions

Taking together, our results suggest that carbohydrate intake above 48% of daily energy intake may contribute to lower SMM and FFM in GG genotype carriers, but AG heterozygotes may benefit from a diet in which more than 48% of energy comes from carbohydrates. If our result is confirmed in larger populations and in populations with different ethnicity, we may also conclude that GG genotype carriers of BDNF rs10835211 should avoid also dietary protein intake higher than 18% and dietary fat intake higher than 30% of their daily energy intake to decrease the risk of metabolic disturbances. In addition, daily fiber intake above 18 g/day by carriers of the BDNF rs10835211 GG genotype may positively affect anthropometric parameters (higher FFM and lower body fat content), but it may also be associated with higher LDL-C and total cholesterol concentrations. Moreover, in AG heterozygotes, it may also have an adverse impact on glucose homeostasis. Our study suggests that there may exist some associations between BDNF gene and diet that may have a significant impact on body weight, obesity-related parameters, glucose metabolism, and lipid profile. Therefore, although the genetic variations and predispositions are inherited, the environmental risk factors are largely modifiable, which may be useful in reducing the risk of developing obesity and its complications.

## Supplementary Information

Supplementary Information.

## Data Availability

The data used and analyzed during the current study are included in this article or are available from the corresponding authors.
