# Supplementary material for: Obesity-related parameters in carriers of some BDNF genetic variants may depend on daily dietary macronutrients intake
Source: Sci Rep. 2023 Apr 21;13:6585. doi: 10.1038/s41598-023-33842-4 (PMC10121660; doi:10.1038/s41598-023-33842-4)
Supplement: Supplementary file 1 — Supplementary Information. [file 41598_2023_33842_MOESM1_ESM.docx]

**Supplemental figure**

Obesity-related parameters in carriers of some BDNF genetic variants may depend on daily dietary macronutrients intake

Urszula Miksza ^1,2^, Edyta Adamska-Patruno ^1,2^, Witold Bauer ^1^, Joanna Fiedorczuk ^1^, Przemyslaw Czajkowski ^1^, Monika Moroz ^1^, Krzysztof Drygalski ^1^, Andrzej Ustymowicz ^3^, Elwira Tomkiewicz ^1^, Maria Gorska ^4^ and Adam Kretowski ^1,2,4^

^1^ Department of Nutriomics, Clinical Research Centre, Medical University of Bialystok, Marii Sklodowskiej-Curie 24A, 15-276 Bialystok, Poland

^2^ Clinical Research Support Centre, Medical University of Bialystok, Marii Sklodowskiej-Curie 24A, 15-276 Bialystok, Poland

^3^ Department of Radiology, Medical University of Bialystok, Marii Sklodowskiej-Curie 24A, 15-276 Bialystok, Poland

^4^ Department of Endocrinology, Diabetology and Internal Medicine, Medical University of Bialystok, Marii Sklodowskiej-Curie 24A, 15-276 Bialystok, Poland

1000PLUS Cohort Study Population

(n = 1549)

Participants without any diseases and treatments that might affect the results

(n = 1285)

Participants with available complete results of genetic and clinical measurements

(n = 894)

Participants, who completed the 3-day food intake diaries

(n = 484)

Gene-diet interactions and analysis of the associations between genetic and clinical/anthropometric measurements

**Figure 1.** Study flowchart diagram of the participants' enrolment process.
